# An environmental signature for 323 microbial genomes based on codon adaptation indices

**DOI:** 10.1186/gb-2006-7-12-r114

**Published:** 2006-12-07

**Authors:** Hanni Willenbrock, Carsten Friis, Agnieszka S Friis, David W Ussery

**Affiliations:** 1 Center for Biological Sequence Analysis, BioCentrum-DTU, The Technical University of Denmark, DK-2800 Lyngby, Denmark

## Abstract

**Background:**

Codon adaptation indices (CAIs) represent an evolutionary strategy to modulate gene expression and have widely been used to predict potentially highly expressed genes within microbial genomes. Here, we evaluate and compare two very different methods for estimating CAI values, one corresponding to translational codon usage bias and the second obtained mathematically by searching for the most dominant codon bias.

**Results:**

The level of correlation between these two CAI methods is a simple and intuitive measure of the degree of translational bias in an organism, and from this we confirm that fast replicating bacteria are more likely to have a dominant translational codon usage bias than are slow replicating bacteria, and that this translational codon usage bias may be used for prediction of highly expressed genes. By analyzing more than 300 bacterial genomes, as well as five fungal genomes, we show that codon usage preference provides an environmental signature by which it is possible to group bacteria according to their lifestyle, for instance soil bacteria and soil symbionts, spore formers, enteric bacteria, aquatic bacteria, and intercellular and extracellular pathogens.

**Conclusion:**

The results and the approach described here may be used to acquire new knowledge regarding species lifestyle and to elucidate relationships between organisms that are far apart evolutionarily.

## Abstract 2

The correlation of two methods for estimating codon adaptation indices applied to more than 300 bacterial species shows that codon usage preference provides an environmental signature by which it is possible to group bacteria according to their lifestyle

## Background

Differential codon usage represents an evolutionary strategy to modulate gene expression, and hence mathematical formulations of the codon usage bias have widely been used to predict gene expression on a genomic scale. This is based on the assumption that codon usage bias is correlated with protein levels. Indeed, highly expressed genes have been found almost exclusively to use those codons translated by abundant tRNAs in *Escherichia coli *and budding yeast, whereas genes that are not highly expressed appear to be less biased in their codon usage. The majority of genes (typically in the range of 90%) are not highly expressed, and the codon usage of these genes appears to be more strongly influenced by mutations than by selection during the course of evolution [1].

Based on these observations, several approaches to measuring codon usage have been proposed to predict the level of protein expression, such as the frequency of optimal codons [2], the codon preference statistic [3], the codon adaptation index (CAI) [1], the 'effective number of codons' used in a gene [4], and predicted highly expressed genes [5]. Of these, the CAI has survived the test of time and has now been cited more than 700 times, with 58 citations in 2005 alone. This method is based on a known set of 27 highly expressed *E. coli *genes [6], from which a codon bias signature was deduced that was most likely to be efficient for translation. This bias was then used to derive codon adaptation indices for all genes in *E. coli*.

Although the first species examined - namely *E. coli *and *Saccharomyces cerevisiae *- provided strong evidence of high translational codon usage bias, recent studies have reported on bacterial species with little codon usage bias [7,8], often species with extreme AT or GC content. In these studies, whole genome information was used to obtain a universal CAI, applying a mathematical measure to derive the most dominant codon bias based on the codons from all potential open reading frames from a genome. This CAI, which ignored the codon usage of experimentally determined highly expressed genes, demonstrated that codon bias, as such, is not necessarily translational nor correlated with gene expression, especially in slow growing bacteria [8]. Consequently, it is not trivial to deduce and compare codon usage biases across a vast range of bacterial species available in sequence databases, including species rich in AT or GC, and to the best of our knowledge this type of large-scale comparison has not previously been conducted.

Although an early report found little correlation between mRNA and protein concentration, the correlation was considerably greater for highly expressed genes [9], and a recent study found a significant relationship between protein levels and mRNA levels in yeast [10]. Consequently, microarray gene expression data are useful for confirming predicted highly expressed genes, as a substitute for protein levels.

Here, we calculate and compare a translational CAI (tCAI) based on that proposed by Sharp and Li [1] with a purely mathematical dominant CAI (dCAI) [8] for 318 bacterial and 5 fungal genomes for which full sequences are deposited in Genbank and available from the Genome Atlas Database (version 19.1) [11]. We compare the ability of both types of CAI to estimate the translational codon bias of an organism and show that codon usage preferences provides an environmental signature by which it is possible to group bacteria according to lifestyle. Furthermore, we examine how well each CAI measure correlates with microarray gene expression data for six selected organisms and show that the tCAI measure is generally better than dCAI in predicting highly expressed genes.

## Results and discussion

**Figure 1:**
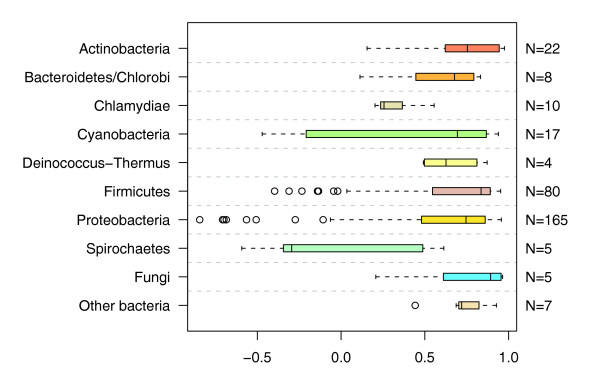
Box plot summarizing correlations between tCAI and dCAI for eight major bacterial phyla and fungi. The group 'Other bacteria' comprises a number of minor bacterial phyla (Aquificae, Chloroflexi, Fusobacteria, Planctomycetes, Acidobacteria, and Thermotogae) that could not meaningfully be included in any of the other categories. The box plot illustrates the median correlations of each group as well as upper and lower quartiles. The numbers on the right side of the figure specifies the number of genomes included in each group. dCAI, dominant codon adaptation index; tCAI, translational codon adaptation index.

The two types of CAI were calculated for all genes in 318 bacterial strains and fungal genomes, and the correlations between the derived tCAI and dCAI values are illustrated for eight different bacterial phyla, with any remaining bacterial species grouped into 'Other bacteria', and fungi depicted separately (Figure 1). For most groups, the correlation between the two CAI measures is high (median above 0.5). Only for chlamydiae and spirochaetes are the median correlations below 0.5, indicating that the dominating codon biases are not translational for most of the species included in these groups. However, it is not surprising that there appears to be little selection for strong tCAI bias in these genomes because most of the bacteria in both of these phyla have slow replication times. Presumably, fast-replicating bacteria have optimized their replication machinery as opposed to slow-replicating bacteria, for which other factors might be more important [7,8,12]. Consequently, we were able to confirm a significant relationship between the level of translational codon adaptation and replication time across the entire range of genomes (Spearman's rank correlation, rho about 0.46) using the number of 16S rRNAs as an indirect measure of doubling time, as previously suggested [13], since the number of 16S rRNAs indirectly influence replication times [14].

**Figure 2:**
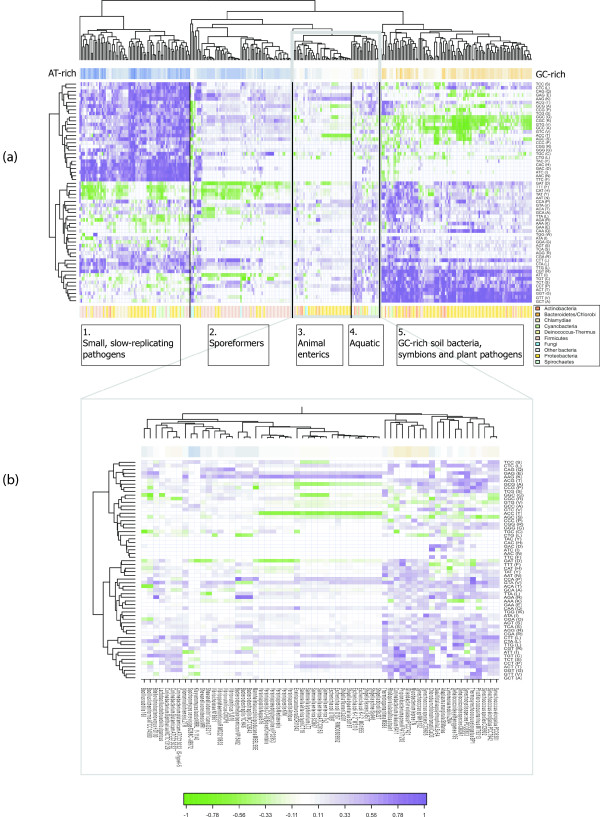
Two-dimensional cluster analysis of differential codon preferences for tCAI and dCAI. The differences in relative adaptiveness of each codon (w_ij _for tCAI minus w_ij _for dCAI) for each Genbank entry were clustered into two dimensions, one clustering codons and the other clustering Genbank entries. The clustering was performed as a hierarchical cluster analysis using Euclidian distances and complete linkage. Codons preferred relatively more by dCAI are red, whereas codons preferred relatively more by tCAI are green. Equal preference is indicated by white. **(a) **Entire dendrogram. The five major regions are indicated and microbial names are replaced by a color bar reflecting each microbe's phylum. **(b) **Zoom of the third and fourth regions. Weights not considered: start codon 'ATG' and stop codons 'TGA', 'TAG' and 'TAA'. dCAI, dominant codon adaptation index; tCAI, translational codon adaptation index.

Next, the codon preferences, which are measurable by the relative adaptiveness of each codon (w_ij_), were compared between tCAI and dCAI and the difference (w_ij _for tCAI minus w_ij _for dCAI) was used for cluster analysis of all 318 bacterial strains and the five fungal genomes (Figure 2a; also see Additional data file: 1, additionally available at our website [15]). Figure 2a shows a clear separation into several clusters with AT-rich bacteria towards the left and GC-rich bacteria towards the right, whereas bacteria with intermediate base composition are in the middle. This is also reflected in the clustering of codons, which are separated into two distinct clusters in which either a codon preference for A/T (lower half) or G/C (upper half) in the third position for dCAI is evident (GC3/AT3 skew dominates over translational bias). However, although the AT content appears to be a significant factor in the clustering, merely ordering by AT content does not yield the same highly distinguishable clusters. Consequently, the correlation between the level of translational codon adaptation (measured by the correlation between tCAI and dCAI) and the genomic AT content was indeed very low but still significant (rho about -0.14, *P *value about 0.015), supporting the minor although unmistakable correlation between AT content and clustering order visible in Figure 2a. Furthermore, from the color bar in Figure 2a, indicating the phylogeny of each microbe, we observe that the clustering is not related to known phylogenetic relationships based on sequence homology. Although smaller clusters of microbes of the same bacterial species are indeed observed, this is perhaps not surprising because genomes of the same species would be expected to have essentially the same codon usage preferences. However, microbes from the same phylum are not clustered but rather are scattered throughout the figure, while many clusters contain organisms that are quite far apart phylogenetically.

The middle area of Figure 2a appears most diverse and can be divided into three distinct regions (ignoring a few smaller clusters on its left side). This division results in a total of five distinct regions, as illustrated in Figure 2a. Figure 2b provides a zoom of the third and fourth region from the left. The third region consists mainly of 'enterics' (intestinal bacteria) living in the human intestine (for example, *Escherichia*, *Shigella*, *Salmonella*, *Bacteroides*), the fly intestine (*Yersinia pestis*), and the animal intestine (*Yersinia pseudotuberculosis*). The yeast genome, *S. cerevisiae*, clusters with the enterics. Although fungi are clearly quite distant from bacteria phylogenetically, both can be relatively fast replicating and hence would face the same selective pressure on codon usage. Moreover, *Kluyveromyces lactis *also groups with the enterics, including *E. coli *K-12, with whom it is often grown together in fermentors to produce chymosin (rennet) on a commercial scale, reflecting similar preferences on growth environment.

The fourth region mostly consists of bacteria living in aquatic environments such as marine waters (*Thermotoga maritima*, *Prochlorococcus marinus*, *Desulfotalea psychrophila*, *Synechococcus species*), groundwater (*Dehalococcoides*), freshwater (*Synechococcus elongatus*), and hot springs (*Thermosynechococcus elongatus*). Although other *P. marinus *strains cluster in the first region, strain MIT9313 is low-light-adapted and has almost as many strain-specific genes as it has genes in common with its high-light-adapted relative, strain MED4 [16], which reflects the differing environmental preferences of the two strains.

Looking at the remaining regions in Figure 2a, we observe that the first (left-most) region consists of slow-growing intracellular pathogens (*Mycoplasma*, *Rickettsia*, and *Chlamydia*, among others) and other small pathogens (*Bartonella*, *Helicobacter*, *Ehrlichia*, and *Campylobacter*), mostly with genome sizes less than or close to 1 megabase (Mbp). The content of this region reflects the observation that many organisms with reduced genomes have very low GC content and supports the speculations that there is a selective pressure in this group of bacteria to lower the nitrogen requirement for DNA synthesis [17] by adapting the codon usage to favor codons with more As and Us. The second region mainly consists of spore formers, including Gram-positive bacteria. Many of the bacteria in this region can replicate quite rapidly, and exhibit other evidence of selective pressure for optimization of the genome for quick replication on demand. For example, the *Vibrio *(a Gram-negative, non-spore-former) and *Bacillus *(a Gram-positive spore-former) cluster close together; and they have the largest number of rRNAs and tRNAs out of several hundred bacterial genomes sequenced so far. Finally, the fifth (right-most) region mainly consists of soil bacteria, soil symbionts and plant pathogens, as well as a few mammalian pathogens. Among additional bacteria in this region, we found an intercellular pathogen, *Brucella melitensis*, that may have evolved from soil and plant associated bacteria [18] and a pathogen, *Wolinella succinogenes*, in which several soil-related genes have been identified [19]. Thus, we find that, upon closer inspection, apparently misplaced genomes in a region may reflect similar shared ecologic niches in the past.

By the above described approach, we were able to divide the organisms into three overall groups reflective of the genomic AT/GC content as previously demonstrated, based on distances between binarized codon weights from dCAI [7]. However, rather than merely discriminating between classes of lifestyle in terms of mesophily, thermophily and hyperthermophily - as previously shown based on either amino acid composition [20,21] or by codon usage [7] - we obtained an environmental signature based on differences in codon weights between evolutionary more dominant codons and codons preferred by the translational machinery. Consequently, we demonstrate that differences in codon usage bias by tCAI and dCAI provide an environmental signature by which it is possible to group bacteria into environmental groups, such as soil bacteria, enterics, sporeformer, and intracellular pathogens. Moreover, this environmental signature does not reflect already known phylogenetic relationships, and as such the approach described above is not intended to replace or extending the existing methods in phylogeny that are based on sequence homology. These results build on a previous finding that GC content of microbial communities is influenced by the environment [22].

### Prediction of highly expressed genes

tCAI is a 'forced' measure of translational bias, whereas dCAI is a measure of the most dominating bias for an organism independently of the type of bias (GC skew bias, strand bias, and so on). For this reason, the correlation between these two measures is a simple and intuitive yet strong indication of whether the most dominating bias is translational, and consequently of how well the dCAI values explain gene expression. In this sense, it is not surprising that the correlation between the two CAI measures also gives an indication of how well tCAI explains gene expression levels. This trend holds true at least for the six organisms for which we compared CAI values with microarray data, where the correlations between the two CAI measures are significantly correlated with the degree of how well tCAI correlates with gene expression (rho = 0.6).

**Figure 3:**
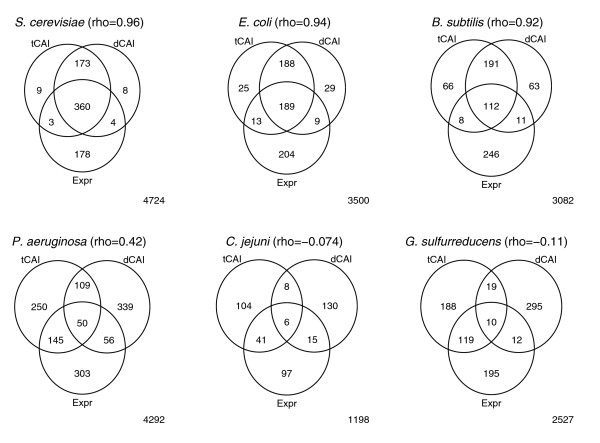
Venn diagram evaluating the prediction of highly expressed genes (tCAI and dCAI) by comparison with microarray gene expression data (Expr). The overlap between genes with top 10% tCAI, dCAI, and Expr values are pictured as overlapping circles, in which the number of genes found by either method is given. The organisms are sorted in order of decreasing correlation (rho) between tCAI and dCAI values based on all genes in each organism. For organisms with high correlation (high rho) between tCAI and dCAI values, most genes are predicted as highly expressed by both measures. Moreover, these predictions overlap significantly with genes found to be highly expressed experimentally by microarrays. For organisms with low correlation (low rho) between tCAI and dCAI values, few genes are predicted as highly expressed by both measures. Here, more genes predicted as highly expressed by tCAI are found to be highly expressed by microarrays than for dCAI. dCAI, dominant codon adaptation index; tCAI, translational codon adaptation index.

To further analyze and compare genes predicted as being highly expressed by tCAI versus genes having extreme codon bias according to dCAI values and versus the highly expressed genes estimated by microarray analysis, the overlap between the top 10% genes was found and visualized in Venn diagrams (Figure 3). For both *S. cerevisiae *and *E. coli *there is good overlap of all three circles; that is, many of the same genes with high tCAI also have high dCAI values, and furthermore these genes are also found to be highly expressed in microarray experiments. For *Bacillus subtilis*, a smaller but similar trend is evident. For the remaining bacteria, a significantly higher number of genes with high expression values (microarray data) overlap with genes with high tCAI values than with genes having high dCAI values. An investigation of the functional categories to which the dCAI reference genes (top 1% of genes) belonged revealed that for *S. cerevisiae*, *E. coli *and *B. subtilis*, a significant fraction of ribosomal proteins were included, whereas for *Pseudomonas aeruginosa*, *Campylobacter jejuni *and *Geobacter sulfurreducens*, no ribosomal proteins where found among dCAI reference genes. This is in agreement with the ribosomal criterion defined by Carbone and coworkers [7], which states that that ribosomal proteins have significantly higher dCAI values than other protein encoding genes in translationally biased organisms. Thus, organisms having few or no ribosomal proteins among dCAI reference genes exhibit little translational codon usage bias as compared with organisms having many ribosomal proteins among dCAI reference genes.

The above comparison of microarray data with tCAI values demonstrates that even for organisms that are evolutionarily far from *E. coli *(for which the bacterial reference set of highly expressed genes was derived), it is possible to predict highly expressed genes by their tCAI values even when the most dominating bias in an organism is not translational, by comparing codon usage for each gene to that of genes in the reference set of highly expressed genes using tCAI. This demonstrates that although the assumption that the same 27 genes are highly expressed in all bacteria may not be entirely true, the codon usage pattern for these genes do provide a useful signature for predicting highly expressed genes. However, the level of confidence decreases with decreasing levels of translational codon adaptation in the dominating codon usage biases (as estimated from the correlation between tCAI and dCAI). Thereby, better performance was obtained than by employing merely the most dominating codon usage bias identified by dCAI, especially for organisms for which translational bias is not dominant (as also observed by Carbone and coworkers [7]); in the latter case, dCAI would not be useful for predicting gene expression at all.

## Conclusion

It was previously postulated that fast-growing bacteria share codon usage preferences because they have more abundant and similar tRNAs [12]. Here, we offer a biological explanation by showing a clear relationship between environment and similarities in codon usage biases. Specifically, differences in codon preferences of translational codon adaptation and dominant codon adaptation provide an environmental signature by which it is possible to divide bacteria into groups representing different lifestyles, such as soil bacteria and symbionts, enterics, aquatic bacteria, spore formers, and small intercellular and extracellular pathogens.

Moreover, our study confirms - across a wide range of bacteria and fungi - that the observed variations in correlation between codon adaptation and gene expression are related to differences in replication times. For organisms with low correlations between tCAI and dCAI, the dominant codon bias is not translational, and consequently the dCAI values do not reflect translational bias. Nonetheless, comparisons of microarray data with tCAI values indicate that this codon adaptation index is still useful for predicting a set of highly expressed genes, although the level of confidence decreases along with the magnitude of the translational bias.

## Materials and methods

All Genbank entries of completely sequenced genomes were taken from version 19.1 (26 May 2006) of the Genome Atlas Database [11].

### Gene expression data

Gene expression data for *E. coli *were downloaded from Gene Expression Omnibus database [23] (GEO); GSM18261 [24], and gene expression data for *C. jejuni *(42°C reference experiments) [25] and *P. aeruginosa *MHH0122 [26] were provided by the authors. For *S. cerevisiae*, preprocessed expression data were downloaded from GEO for two yeast strains, namely BY4741 (samples GSM6711, GSM6712, and GSM6713) [27] and BY4716 (samples GSM35294, GSM35295, and GSM35296) [28], both of which are derived from the S288C strain.

All raw data were normalized with qspline [29] and expression indices were estimated [30]. BY4741 expression data were log transformed and all preprocessed *S. cerevisiae *data were re-normalized by qspline together with 179 additional expression profiles for the same Affymetrix YG-S98 chip downloaded from GEO. For *C. jejuni *the median of normalized data was used, and for *S. cerevisiae *the mean of the two strain medians was used.

Additional processed expression data were downloaded from ArrayExpress for *G. sulfurreducens *(ATCC 51573: GGS23_BR2_2S_12679025) [31] and *B. subtilis *(25866GENEPIX25866) [32]. No further treatment of these data was conducted.

### Translational codon adaptation index

The CAI measure of translational adaptation is an updated version of the original codon adaptation index reported by Sharp and Li [1], and in the following we refer to this CAI measure as the 'translational codon adaptation index' (tCAI). Although Sharp and Li, in their original work from 1987, were forced by lack of data to assume a background codon usage corresponding to equal usage of the synonymous codons for any given amino acid, we now have vast libraries of complete genomic sequences available. Consequently, we calculate the relative synonymous codon usage (RSCU) for each organism by comparing the codon distribution from a set of highly expressed genes with a background distribution estimated from the codon usage of all coding regions in the genome as annotated in the Genbank entries:

RSCUij=Xij∑j=1niXij×∑j=1niYijYij
 MathType@MTEF@5@5@+=feaafiart1ev1aaatCvAUfeBSjuyZL2yd9gzLbvyNv2Caerbhv2BYDwAHbqedmvETj2BSbqee0evGueE0jxyaibaiKI8=vI8tuQ8FMI8Gi=hEeeu0xXdbba9frFj0=OqFfea0dXdd9vqai=hGuQ8kuc9pgc9s8qqaq=dirpe0xb9q8qiLsFr0=vr0=vr0dc8meaabaqaciGacaGaaeqabaqadeqadaaakeaacaqGsbGaae4uaiaaboeacaqGvbWaaSbaaSqaaiaabMgacaqGQbaabeaakiabg2da9maalaaabaGaaeiwamaaBaaaleaacaqGPbGaaeOAaaqabaaakeaadaaeWaqaaiaabIfadaWgaaWcbaGaaeyAaiaabQgaaeqaaaqaaiaabQgacqGH9aqpcaaIXaaabaGaaeOBamaaBaaameaacaqGPbaabeaaa0GaeyyeIuoaaaGccqGHxdaTdaWcaaqaamaaqadabaGaaeywamaaBaaaleaacaqGPbGaaeOAaaqabaaabaGaaeOAaiabg2da9iaaigdaaeaacaqGUbWaaSbaaWqaaiaabMgaaeqaaaqdcqGHris5aaGcbaGaaeywamaaBaaaleaacaqGPbGaaeOAaaqabaaaaaaa@54C0@

Here, X_ij _represents the number of observations of the jth codon for the ith amino acid in the set of highly expressed genes, whereas Y_ij _is the corresponding number of observations in the background set. Furthermore, n_i _is the number of codons for the ith amino acid, with RSCU_i,max _being the highest number from the vector of RSCU_i _= (RSCU_ij _= 1 ... RSCU_ij _= n_i_).

The relative adaptiveness of a codon (w_ij_) is calculated as follows:

W_ij _= RSCU_ij_/RSCU_i,max_

Subsequently, CAIs for individual coding regions were obtained as follows:

CAI=exp⁡1L∑k=1Llnwk
 MathType@MTEF@5@5@+=feaafiart1ev1aaatCvAUfeBSjuyZL2yd9gzLbvyNv2Caerbhv2BYDwAHbqedmvETj2BSbqee0evGueE0jxyaibaiKI8=vI8tuQ8FMI8Gi=hEeeu0xXdbba9frFj0=OqFfea0dXdd9vqai=hGuQ8kuc9pgc9s8qqaq=dirpe0xb9q8qiLsFr0=vr0=vr0dc8meaabaqaciGacaGaaeqabaqadeqadaaakeaacaqGdbGaaeyqaiaabMeacqGH9aqpciGGLbGaaiiEaiaacchadaWcaaqaaiaaigdaaeaacaqGmbaaamaaqadabaGaaeiBaiaab6gacaqG3bWaaSbaaSqaaiaabUgaaeqaaaqaaiaabUgacqGH9aqpcaaIXaaabaGaaeitaaqdcqGHris5aaaa@4467@

Where L is the number of codons in a given gene.

In order to identify a set of constitutively highly expressed genes for each of the 318 bacterial genomes analyzed in this work, the reference set of 27 very highly expressed *E. coli *genes originally compiled by Sharp and Li [6] was aligned at the protein level against all genes annotated in the Genbank entry for each genome using BLASTP version 2.2.9 [33]. For each of these very highly expressed genes, the gene with the best alignment was added to a set of very highly expressed genes if it had an E value below 10^-6 ^(the absolute minimum accepted only if an alignment with a better score could not be identified), and these were used as a reference set for the given organism. Similarly, for the five fungal genomes, we used the reference set of very highly expressed *S. cerevisiae *genes identified by Shartp and coworkers [34], removing the second ribosomal protein 51 gene (rbs51B), resulting in a list of 37 genes.

By this procedure, we were able to construct reference sets containing a minimum of 15 genes for the Firmicute *Clostridium tetani *E88, and a maximum of 27 highly expressed *E. coli *reference genes for 26 Proteobacteria strains. Consequently, bacteria more related to *E. coli *exhibited a higher level of conservation. Thus, the number of identified reference genes ranged from a median of 24 for Proteobacteria to a median of 21 for Actinobacteria. For the fungal genomes, a median of 36 genes was found in the reference sets.

Alternatively, profile hidden Markov models from Pfam [35], representing each of the genes in the reference sets, were used to identify sets of highly expressed reference genes. However, this resulted in a slightly worse performance.

### Dominating codon bias index

A purely mathematical CAI measure was proposed by Carbone and coworkers [8], and in this report we refer to this CAI measure as 'dominant codon adaptation index' (dCAI). It detects the most dominant codon bias in the genome, regardless of whether this bias is translational. The algorithm screens a genome for genes that score the highest values on the CAI scale and selects these as its reference set. For dCAI values, we have used the tool CAIJava available from Carbone and coworkers [8].

### Data treatment

All DNA and protein sequence information was extracted from each Genbank entry. For correlation estimates, we used Spearman's rank correlation [36] to avoid any problems with possible deviations from normality in compared data (for example, log-normal distribution for microarray data). Cluster analysis was based on hierarchical clustering of Euclidian distances by complete linkage.

## Additional data files

Additional data file: 1 contains a supplementary figure, which provides a detailed view of clusters illustrated in figure 2. This is also available at our website [15].

## Supplementary Material

Additional Data File 1 A detailed version of the cluster analysis in Figure 2, providing the full organism names. Click here for file
