# Supplementary figures and images for: An environmental signature for 323 microbial genomes based on codon adaptation indices
Source: Genome Biol. 2006 Dec 7;7(12):R114. doi: 10.1186/gb-2006-7-12-r114 (PMC1794427; doi:10.1186/gb-2006-7-12-r114)

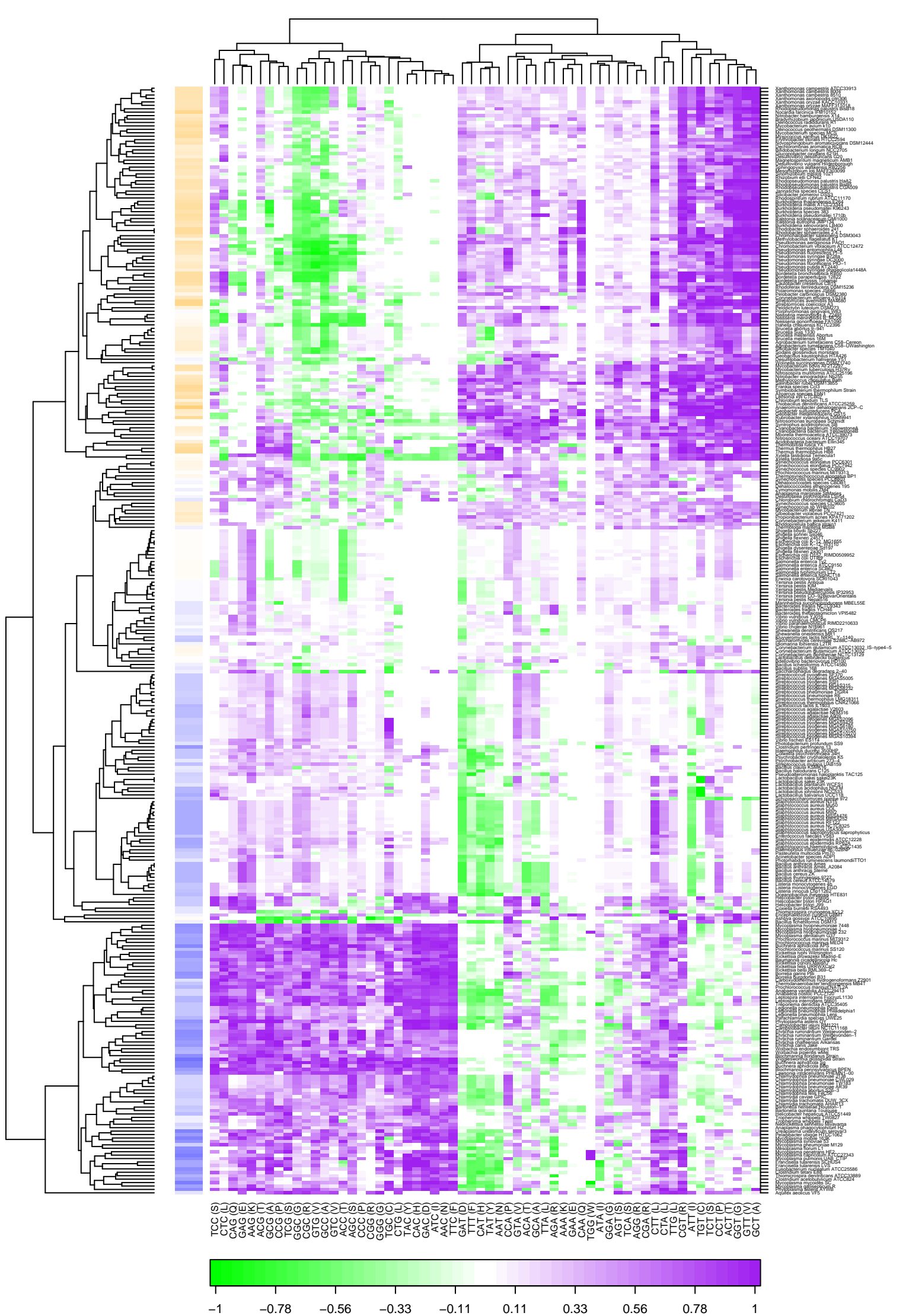

Supplement: Additional Data File 1 — A detailed version of the cluster analysis in Figure 2, providing the full organism names. [file gb-2006-7-12-r114-S1.pdf]
